# Urinary Markers of Tubular Injury in HIV-Infected Patients

**DOI:** 10.1155/2016/1501785

**Published:** 2016-07-17

**Authors:** Temesgen Fiseha, Angesom Gebreweld

**Affiliations:** Department of Clinical Laboratory Science, College of Medicine and Health Sciences, Wollo University, Dessie, Ethiopia

## Abstract

Renal disease is a common complication of HIV-infected patients, associated with increased risk of cardiovascular events, progression to AIDS, AIDS-defining illness, and mortality. Early and accurate identification of renal disease is therefore crucial to improve patient outcomes. The use of serum creatinine, along with proteinuria, to detect renal involvement is essentially to screen for markers of glomerular disease and may not be effective in detecting earlier stages of renal injury. Therefore, more sensitive and specific markers are needed in order to early identify HIV-infected patients at risk of renal disease. This review article summarizes some new and important urinary markers of tubular injury in HIV-infected patients and their clinical usefulness in the renal safety follow-up of TDF-treated patients.

## 1. Introduction 

Renal disease is a common complication of HIV-infected patients, associated with increased risk of cardiovascular events, progression to AIDS, AIDS-defining illness, and mortality (both all-cause and AIDS-related) [1–4]. Moreover, HIV-infected patients have a faster decline in renal function and are at higher risk of progression to end stage renal disease (ESRD), requiring costly renal replacement therapy in the form of dialysis or transplantation [5]. Despite the potential benefit of highly active antiretroviral therapy (HAART), the prevalence of patients with AIDS and subsequently the population at risk for ESRD will continue to increase, suggesting that HIV-related renal diseases will pose a substantial public health and resource problem in the near future [6]. It has been also reported that since the introduction of HAART, renal disease not directly related to HIV has become the predominant cause, reflecting the growing burden of comorbidities in this aging population [7, 8]. Early and accurate identification of renal disease is therefore crucial to facilitate the employment of measures that can slow progression to ESRD and improve patient outcomes.

Current guidelines for the management of HIV-infected patients recommended the assessment of renal disease by using proteinuria and creatinine-based estimated glomerular filtration rate (eGFR) [9]. However, a substantial proportion of HIV-infected patients could have renal diseases in the absence of proteinuria and/or altered glomerular function [10–12]. Furthermore, the use of serum creatinine (SCr), along with proteinuria, to detect renal involvement is essentially to screen for markers of glomerular disease and may not be effective in detecting earlier stages of renal injury. Recent studies have demonstrated that there is also a tubulointerstitial component to renal complications of HIV infection and treatment [13–15]. Importantly, primary tubulointerstitial alterations, even severe, may be missed until they affect the glomerular function. Therefore, more sensitive and specific markers in addition to the traditional markers are needed in order to identify HIV-infected patients with early renal disease who may benefit from interventions known to delay disease progression and prevent complications.

Renal tubular abnormalities are common and a substantial proportion of HIV-infected patients on HAART could have prevalent renal tubular injury in the absence of glomerular defects, probably resulting in a near future decrease in glomerular function and a higher incidence of urinary protein [16–18]. Moreover, the use of tenofovir (TDF) leads to an increased rate of renal tubular injury characterized by increased urinary excretion of tubular proteins, even in patients with normal baseline renal function, and is associated with unrecognized and permanent renal function decline [19–22]. Urinary markers of tubular injury could therefore potentially be useful in the early identification of patients at risk for severe renal tubular disease as well as TDF-induced nephrotoxicity. Several of these tubular markers increase in the urine of HIV-infected patients, even before diagnosis of proteinuria and/or a decrease in glomerular function representing early markers of renal disease [18, 23, 24]. The aim of this review is to summarize some new and important urinary markers of tubular injury associated with early renal damage or dysfunction in HIV-infected patients and their clinical usefulness in the renal safety follow-up of TDF-treated patients.

## 2. Neutrophil Gelatinase-Associated Lipocalin

Neutrophil gelatinase-associated lipocalin (NGAL) is a small, 25-kDa protein that belongs to the lipocalin protein family released from neutrophils and many epithelial cell types including kidney tubular cells. It is representative of the functioning tubular mass and is secreted into the urine as a response to tubular injury [25]. HIV-infected patients had higher median urine levels of NGAL than uninfected patients, and HIV infection was associated with 19% (4–36%) higher NGAL/Cr, suggesting that HIV-infected patients had more extensive kidney injury than uninfected [26]. In HIV-positive outpatients, elevated urinary NGAL/creatinine ratio measurements were found in 67% of patients [27]. Additionally, urinary NGAL is elevated even before the eGFR is reduced, is reflective of injury in parts of the nephron other than the glomerulus, and has independent associations with faster kidney function decline and mortality risk in HIV-infected patients [28, 29].

Urinary NGAL was significantly higher in patients with HIVAN compared with HIV-infected patients without renal disease and HIV-negative controls [30, 31]. The excretion of urinary NGAL was much higher in patients with biopsy-proven HIVAN than in HIV-positive patients and negative controls with other forms of CKD [30, 32]. In HIVAN, urinary NGAL was upregulated 5-fold and 11-fold in comparison with HIV-positive patients with CKD of non-HIVAN etiology and that of patients without kidney disease, respectively [32]. Markedly elevated levels of urinary NGAL was also found in HIVAN patients with relatively preserved kidney function (SCr < 2, eGFR range 56.22–117.35) and limited proteinuria (range 0.0–3.0 mg/mL), suggesting that NGAL can be expressed early in the course of progressive renal failure due to HIVAN [32]. Moreover, urinary NGAL showed no correlation with SCr, eGFR, or proteinuria in HIVAN, and the receiver operator characteristic (ROC) curve for urinary NGAL indicated excellent diagnostic utility to detect HIVAN, demonstrating aid in the noninvasive diagnosis of HIVAN and screening for HIVAN-related tubular damage [30, 32]. Thus, urinary NGAL appears to be robustly expressed in HIVAN and may be useful to monitor the presence of renal tubular injury and consequently distinguish HIVAN from other forms of CKD or other glomerulopathies presenting in the HIV patient.

## 3. Alpha-1 Microglobulin

Alpha-1 microglobulin (A1M) is a small molecular weight protein (27 kDa) present in various body fluids. In the healthy kidney, it passes freely through the glomerular membranes, and about 99% is reabsorbed and catabolized by the proximal tubular cells. Increased A1M in urine can therefore be an early sign of renal damage, primarily on the proximal tubules [33]. Urinary excretion of A1M was significantly higher in HIV-infected patients compared with uninfected patients and was associated with HIV infection [34, 35]. HIV infection was associated with 136% higher urine A1M levels and 1.5-fold or 51% prevalence of detectable A1M. HIV-related factors, including CD4 lymphocyte count < 200 cells/mm^3^ and history of AIDS, were also associated with higher urinary A1M levels [34]. Higher urinary levels of A1M have been found in HIV-infected patients with HIVAN compared to patients without renal disease, suggesting that A1M could be used as a predictor of HIVAN [31].

Urinary A1M has been suggested to be the most sensitive parameter to detect kidney tubular dysfunction in HIV-infected patients [36]. Among HIV-infected patients, 54.4% were positive for tubular proteinuria as measured by urinary A1M/Cr, while 12.1% were positive for albuminuria, indicating the presence of early tubular dysfunction [37]. In a cohort of HIV-positive patients, 41% presented tubular proteinuria by assessing A1M and 20% glomerular proteinuria (urinary ACR) [16]. HIV patients with rather than without tubular damage also showed a high urinary excretion of A1M, often in the absence of any sign of proteinuria and/or significant impairment of the glomerular function [18]. These data suggest that the detection of subclinical tubular damage using urinary biomarkers, such as A1M, may allow significant kidney disease to be identified before any overt evidence of glomerular defects can be obtained.

As tubular proteinuria defined by A1M is associated with exposure to TDF [16], urinary A1M has been suggested to be a more sensitive biomarker of tubular dysfunction for assessing TDF-related nephrotoxicity [18, 37, 38]. Urinary A1M was significantly higher in patients with TDF-associated kidney tubular dysfunction compared to patients with normal tubular function [39]. Both the frequency of urinary A1M/Cr and the median urinary A1M/Cr were higher in the TDF group than the treatment-naïve or non-TDF-containing regimens [37]. Additionally, cumulative TDF exposure was associated with incrementally higher A1M levels, whereas time since TDF treatment discontinuation was associated with progressively lower A1M levels, suggesting a significant role for urinary A1M in detecting and monitoring TDF-associated tubular toxicity [35]. Urinary A1M also showed a better diagnostic accuracy as a marker for TDF-induced kidney tubular dysfunction when compared to the classical clinical markers of tubular damage: fractional excretions of phosphate and uric acid [38]. Furthermore, urinary A1M was a predictor of kidney function decline and mortality risk in HIV-infected patients independent of traditional CKD risk factors and albuminuria [34]. Compared to HIV-infected patients with undetectable urine A1M, HIV-infected women in the highest category of A1M/Cr had a 2.1-fold risk of incident CKD, 2.7-fold risk of 10% annual eGFR decline, and 1.6-fold mortality risk, indicating its value for identifying HIV-infected patients at risk of adverse outcomes [34].

## 4. Beta 2-Microglobulin

Beta (*β*) 2-microglobulin (B2-M) is an 11.8 kDa protein produced by cells expressing major histocompatibility complex (MHC) class I. It is filtered freely through the glomeruli of the kidney, and a majority of it is reabsorbed and catabolized by renal proximal tubular cells; thus, measurement of urinary B2-M can serve as a useful biomarker to evaluate renal tubular injury [40, 41]. Urinary excretion of B2-M was significantly higher in HIV-infected patients than in uninfected controls [42, 43], and high urine levels suggested the presence of tubular injury in these infected patients [31]. Urinary B2-M elevation is associated with uncontrolled viremia, boosted protease inhibitor use, hepatitis C, and GFR < 60 mL/minute [44].

High urinary levels of B2-M were found in children with biopsy-proven HIVAN than HIV-infected children without renal disease, which suggested that urinary B2-M excretion might be a useful biomarker for detecting the presence of tubular injury in HIV patients [31, 42]. Urinary B2-M levels were also elevated in HIV-infected children with proteinuria, indicating early proximal tubular injury in these patients [31]. Higher levels of urinary B2-M were also found in HIV-infected patients with rather than without tubular damage prior to the appearance of proteinuria or a decrease in eGFR below 60 mL/min/1.73 m^2^ [18]. Another study also found abnormal urinary B2-M excretion in 11.8% of HIV-infected patients without renal disease, suggesting the importance of urinary B2-M as a biomarker for detecting tubular injury before the emergence of substantial glomerular damage or dysfunction [42].

B2-M was more likely to be elevated among TDF users compared with non-TDF users and it has been suggested that B2-M concentrations in urine may become useful in diagnosis of renal failure and are more sensitive than the standard determination of creatinine in plasma and eGFR in patients treated with TDF [44, 45]. Urinary excretion of B2-M was significantly higher in patients with TDF-associated kidney tubular dysfunction compared to patients with normal tubular function [39, 46], and relative to SCr, it could be a more sensitive marker of renal tubular injury caused by TDF [47]. Urinary B2-M also showed a better diagnostic accuracy for TDF-induced kidney tubular dysfunction as compared to fractional excretions of phosphate and uric acid [38]. Increased urinary excretion of B2-M was also found in a substantial proportion of HIV-infected patients with TDF associated proximal tubular kidney damage or dysfunction, often in the absence of any sign of proteinuria and/or impaired glomerular function [18, 23, 48]. The urinary excretion of B2-M was significantly higher in TDF (+) patients than that in TDF (−) patients [37, 47], and urinary B2-M measured within 180 days after initiation of TDF-containing ART predicted renal dysfunction related to long-term TDF use [49]. High urine B2-M was also observed in 71% of patients on TDF and the mean urine B2-M for the TDF group was significantly higher at 12 weeks than the controls [50]. Thus, B2-M is a potentially suitable screening marker in HIV-infected patients and monitoring urinary B2-M should be useful in early detection of TDF nephrotoxicity [38].

## 5. N-Acetyl-Beta-D-Glucosaminidase

N-acetyl-beta-D-glucosaminidase (NAG) is a hydrolytic lysosomal enzyme found predominantly in proximal tubule. It has been demonstrated as a useful marker of renal tubular impairment in various conditions involved with renal injury or dysfunction [51]. Urinary concentrations of NAG were found to be increased in HIV-infected patients [24, 37, 38] and to correlate with urinary ACR and PCR [24]. The average urinary excretion of NAG was significantly higher in HIV-infected patients than in the HIV-negative controls [42]. Elevated levels of NAG were associated with history of proteinuria and GFR < 60 mL/minute [44]. In addition, HAART vintage of more than 2.5 years was statistically associated with increased urinary NAG in HIV-infected patients [52].

In HIV-infected patients, urinary excretion of NAG was significantly higher in those with rather than without tubular damage [18]. In one study, tubular damage defined by increased urinary NAG levels was found in 37.5% of HIV-infected outpatients [52]. An increase in urinary NAG levels was also found in HIV-infected patients even in the absence of proteinuria or significant impairment of glomerular function, indicating its ability to identify tubular damage prior to the emergence of overt glomerular disease [18]. Urinary excretion of NAG was also significantly higher in patients with TDF-associated kidney tubular dysfunction compared to patients with normal tubular function [18, 39]. Higher levels of urinary NAG/Cr was also found in HIV patients on ART (regardless of whether it included TDF) compared with the ART-naive patients, while there were no significant differences in urinary ACR (a marker of glomerular disease) values [24]. However, future studies are needed to explore urinary NAG in identifying patients at greater risk for developing HIVAN or TDF-associated tubulopathy and close monitoring of renal function.

## 6. Kidney Injury Molecule-1

Kidney injury molecule-1 (KIM-1) is a transmembrane protein and its expression is not measurable in normal proximal tubule cells but is markedly upregulated with injury/dedifferentiation [53]. It has been suggested that its presence in the urine is highly specific for kidney injury and may serve as a useful biomarker for renal proximal tubule injury facilitating the early diagnosis of the disease [53, 54]. Urine concentrations of KIM-1 were higher in HIV-infected patients as compared with HIV-uninfected participants, and urinary KIM-1 concentrations were 12% higher in HIV-infected individuals [26, 29]. In the above study, HIV infection was associated with 16% (6–27%) higher urine KIM-1/Cr ratio, demonstrating usefulness of urinary KIM-1 as a marker for screening and quantifying kidney injury in HIV-infected individuals [26].

Higher concentration of KIM-1/Cr was observed in HIV-infected patients receiving antiretroviral therapy as compared to healthy individuals, although the difference was not statistically significant [55]. Cumulative exposure to TDF was independently associated with 3.4% higher KIM-1, but not with albuminuria; suggesting that urinary KIM-1 may be more sensitive than albuminuria for detecting toxicity from TDF and other medications [56]. Urinary KIM-1 was also found to have strong and independent associations with both linear and dichotomized outcomes of kidney function decline in HIV-infected patients. Compared with the lowest tertiles, the highest tertiles of KIM-1 were independently associated with both faster eGFRcys and eGFRcr decline, suggesting that novel urine markers of kidney injury, such as KIM-1, detect risk for subsequent declines in kidney function among HIV-infected patients [29]. Highest tertiles of KIM-1 were also associated with higher risk of mortality in HIV-infected patients [28]. All these data suggest that KIM-1 is a promising marker for early tubular damage in patients with HIV infection; however, further studies are required to document the clinical utility of urinary KIM-1 for early renal disease in these patients.

## 7. Retinol Binding Protein

Retinol-binding protein (RBP) is a low molecular weight protein (21 kDa) that transports retinol (vitamin A alcohol) from the liver to peripheral tissues. It passes freely through glomerular membranes and is reabsorbed by renal proximal tubules cells where it is catabolized, so an increase in the urinary excretion of RBP indicates proximal tubule injury and/or impaired proximal tubular function [57, 58]. High levels of urine RBP suggest the presence of tubular injury in HIV-infected patients [31]. High urinary levels of RBP were found in children with biopsy-proven HIVAN, relative to the samples from HIV-infected children without renal disease and the normal range control values, demonstrating its potential value to be used as a useful biomarker reflecting the presence of tubular injury [31]. The urinary RBP levels were also significantly elevated in HIV-infected children with trace proteinuria, suggesting that proximal tubular injury is an early event in the pathogenesis of HIV-related renal diseases [31].

Urinary excretion of RBP was significantly higher in HIV-infected patients than in the HIV-negative controls, even in patients without renal disease [42]. In this study, over 80% of HIV-infected patients without overt renal disease have evidence of glomerular permeability defects or tubular dysfunction, and urinary RBP together with albuminuria are the most sensitive and reliable early marker of these abnormalities [42]. In one study, abnormally high urinary RBP/Cr was observed in 54.3% of HIV-positive patients with normal estimated filtration rate [59]. Another study, which recruited HIV patients with SCr levels < 1.70 mg/dL and dipstick-negative proteinuria, also showed that the level of RBP was increased in the urine of HIV-infected patients compared to healthy controls [24]. In this study, the proportion of patients with tubular dysfunction (increased urinary RBP/Cr and/or NAG/Cr) was considerably higher than the proportion with an increase in urinary ACR (a marker of glomerular disease) and urinary PCR (a standard clinical screening test for kidney pathological states) in all groups [24].

RBP is also a promising biomarker for monitoring renal tubular function in patients receiving TDF and for distinguishing patients with normal tubular function or mild tubular dysfunction from those with severe renal tubular disease and Fanconi syndrome [27]. Urinary RBP defined renal tubular dysfunction was found in 22.5% of patients receiving TDF-containing ART [60]. Urinary RBP/Cr was also higher in the patients exposed to TDF than either the ART-naive or those with non-TDF [24]. Moreover, mean RBP levels in patients on TDF both with decreased and normal baseline GFR were significantly higher than in controls [45]. Abnormal urinary RBP/Cr levels were also found in a high proportion of patients on TDF-containing HAARTs who presented normal estimated filtration rate [59]. This indicates that RBP concentrations in urine may become useful in diagnosis of renal failure and is more sensitive than the standard determination of creatinine in plasma and eGFR in patients treated with TDF- containing HAARTs [45]. Furthermore, renal tubular dysfunction defined by 5-fold elevation in urinary RBP/Cr was associated with significantly lower bone mineral density of the spine, indicating its potential value in identifying HIV-infected patients at increased risk of TDF-associated bone mineral density loss [60].

## 8. Liver-Type Fatty Acid Binding Protein

Liver-type fatty acid binding protein (L-FABP) is a low molecular weight (15 kDa) intracellular carrier protein that is expressed in the renal proximal tubule and liver. In renal disease L-FABP gene expression in the kidney was upregulated and its urinary excretion was found to correlate with the severity of tubulointerstitial injury, reflecting stresses on the proximal tubules [61]. Higher concentration of L-FABP/Cr was observed in HIV-infected as compared to healthy individuals [55]. Urinary excretion of L-FABP was significantly elevated in patients with HIVAN compared to HIV-positive patients with non-HIVAN glomerulopathies [30]. The ROC curve for urinary L-FABP indicated excellent diagnostic utility to detect HIVAN, indicating that urinary L-FABP could be used as a noninvasive clinical marker of HIVAN [30].

Higher concentrations of urinary L-FABP/Cr were also found in HIV/HCV coinfected patients as compared to the HIV-monoinfected individuals and to healthy subjects. Furthermore, patients receiving the regimen based on EFV had higher levels of L-FABP/Cr compared to individuals treated with other regimens and to healthy controls [55]. All the above data suggest that L-FABP is a promising marker for early tubular damage in patients with HIV infection; however, further studies are required to document its clinical utility for early detection of renal disease in these patients.

## 9. Cystatin C

Cystatin C (CysC), a cysteine protease inhibitor constantly produced by all nucleated cells, is a low molecular weight (13 kD) basic protein freely filtered by the kidney glomerulus and is reabsorbed by the tubules, where it is almost totally catabolized [62]. Individuals infected with HIV had substantially worse kidney function when measured by cystatin C level when compared with HIV-negative controls and suggested that cystatin C measurement could be useful to identify HIV patients at higher risk for renal and CV disease, and of mortality [63, 64]. Similar to the serum CysC, increased urinary CysC has been recognized as a marker of renal tubular dysfunction [65]. Urinary CysC concentration has been found to be increased in HIV subjects [42] and to correlate with GFR and B2-M in patients with HIV infection [66]. Urinary CysC/Cr also correlates with urinary RBP/Cr, suggesting that CysC may be better markers of specific forms of proximal tubular dysfunction in these patients [27]. It has been also suggested that CysC may be useful as a marker of renal function in HIV-infected patients, independently of ongoing inflammation or viremia [66].

Higher levels of urinary CysC were found in patients with established HIV-associated renal diseases than in patients without proteinuria or trace proteinuria [67]. CysC levels are elevated in persons with HIV infection and remain so even after HIV RNA levels are suppressed with antiretroviral therapy [68]. In a population of GFR, sex and age-matched TDF-naive patients without symptoms of proximal tubule injury, the urinary CysC/Cr was under the detection limit for all urine specimens. The ROC curve analysis of this study showed that urinary CysC/Cr had a high area under curve (AUC: 0.929) to distinguish a Fanconi positive patient from a Fanconi negative one, allowing to rule out a Fanconi syndrome, defects of proximal tubular transport function. Finally, the study suggested that the measurement of the urinary CysC/Cr on a single urine voiding is a simple and efficient marker to detect or rule out proximal tubule dysfunction induced by TDF; thus, it should be used for the safety follow-up of nucleotide reverse transcriptase inhibitor-treated patients [69].

## 10. Conclusions

The use of creatinine-based estimates of renal function and proteinuria to detect renal involvement is essentially to screen for markers of glomerular disease and may not detect abnormalities in the tubular function. Urinary excretions of the tubular proteins are significantly higher in HIV-infected patients compared with the uninfected controls and in the patients with rather than without tubular injury. The urinary excretion of these tubular proteins increase even before the diagnosis of proteinuria and/or a decrease in glomerular function, indicating that renal tubular injury is an early event in patients with HIV and tubular proteins could be useful for evaluating early renal involvement [18]. Exposure to TDF is associated with subclinical renal tubular injury, inducing increased urinary excretion of the tubular proteins, even in patients without preexisting glomerular defects, suggesting a significant role for these tubular proteins in the safety follow-up of TDF-treated patients. Collectively, these data suggest that the tubular proteins, such as NGAL, A1M, B2-M, and RBP are early, sensitive markers of tubular damage or dysfunction, and their measurements in urine potentially allow early identification of HIV-infected patients at risk of subclinical renal injury or TDF-associated nephrotoxicity. Although increased fractional excretions of phosphate, uric acid, and glycosuria are established markers of proximal tubule dysfunction and are easy to screen for; they are less sensitive tests than tubular protein excretions and have been suggested to be the most appropriate alternatives when these are not available [70, 71].

## Abbreviations

A1M: Alpha-1 microglobulin
B2-M: Beta (*β*) 2-microglobulin
Cr: Creatinine
CysC: Cystatin C
eGFR: Estimated glomerular filtration rate
HIVAN: HIV-associated nephropathy
KIM-1: Kidney injury molecule-1
L-FABP: Liver-type fatty acid-binding protein
NAG: N-acetyl-beta-glucosaminidase
NGAL: Neutrophil gelatinase-associated lipocalin
RBP: Retinol-binding protein
TDF: Tenofovir disoproxil fumarate.
